# Novel pebbles in the mosaic of autoimmunity

**DOI:** 10.1186/1741-7015-11-101

**Published:** 2013-04-04

**Authors:** Carlo Perricone, Nancy Agmon-Levin, Yehuda Shoenfeld

**Affiliations:** 1 The Zabludowicz Center for Autoimmune Diseases, Sheba Medical Center, Tel Hashomer 52621, Israel; 2 Reumatologia, Dipartimento di Medicina Interna e Specialità Mediche, Sapienza Università di Roma, Rome, Italy; 3 Incumbent of the Laura Schwarz-Kip Chair for Research of Autoimmune Diseases, Tel-Aviv University, Tel Aviv, Israel

**Keywords:** Autoimmunity, Autoantibodies, Autoimmune/inflammatory syndrome induced by adjuvants (ASIA), Systemic lupus erythematosus, Vitamin D, Adjuvant

## Abstract

Almost 25 years ago, the concept of the ‘mosaic of autoimmunity’ was introduced to the scientific community, and since then this concept has continuously evolved, with new pebbles being added regularly. We are now looking at an era in which the players of autoimmunity have changed names and roles. In this issue of *BMC Medicine*, several aspects of autoimmunity have been addressed, suggesting that we are now at the forefront of autoimmunity science. Within the environmental factors generating autoimmunity are now included unsuspected molecules such as vitamin D and aluminum. Some adjuvants such as aluminum are recognized as causal factors in the development of the autoimmune response. An entirely new syndrome, the autoimmune/inflammatory syndrome induced by adjuvants (ASIA), has been recently described. This is the new wind blowing within the branches of autoimmunity, adding knowledge to physicians for helping patients with autoimmune disease.

## Introduction

Almost 25 years ago the concept of the ‘mosaic of autoimmunity’ [1] was introduced to the scientific community. Since then, it has continuously evolved, with new tiles being added regularly [2], and we are now looking at an era in which the players of autoimmunity have changed names and roles. In this issue of *BMC Medicine*, several aspects of autoimmunity have been addressed suggesting that we are now at the forefront of autoimmunity science.

One of the mainstays of the mosaic concept is that autoimmune diseases (ADs) occur in genetically predisposed individuals [3]. This concept has now been expanded by recent evidence of ‘familial autoimmunity’: not only is there familial aggregation for individual ADs [4,5], but there is also a familial aggregation of diverse autoimmune diseases. This was brought to light by evidence from a systematic review and meta-analysis performed by Cárdenas-Roldán *et al*. [6]. Physicians should be aware that familial autoimmunity is a common finding, especially for some specific disorders, such as autoimmune thyroid disease and systemic lupus erythematosus (SLE), suggesting a stronger shared genetic influence in their development.

Strengthening the theory of a genetic basis is the evidence that ADs occur more often in young people [7]. However, although ADs were thought to be rare in older people, the validity of this assumption has been challenged, and a tendency towards more severe autoimmunity in older people has been noted [8]. A possible explanation for this paradox comes from another characteristic of the disease mosaic, namely, the presence of an abnormal immune response. Vadasz *et al*. [9] suggested that expansion of many protective regulatory mechanisms and especially of peripheral CD4+CD25_high_FoxP3+ T-regulatory cells, is very characteristic in elderly people. It is possible that during aging, an imbalance between thymic and peripheral regulatory T-cell output occurs, with the ratio favoring the peripheral component, which possibly allows a pro-inflammatory response and increases the susceptibility to autoimmunity. Furthermore, in addition to this disruption of adaptive immunity, it has been shown that disruption of the autoimmune response also occurs in the innate immune system [10]. Pollard *et al*. [11] suggested that differences in autoimmune responses are mainly mediated by the dichotomy in Toll-like receptor (TLR) signaling that distinguishes interferon regulatory factor 7-mediated type I interferon production from nuclear factor-kappa B (NFκB)-driven pro-inflammatory cytokine expression. Indeed, TLRs play a crucial role in the activation of both innate and adaptive immunity [12]. By recruiting various protein kinases via several adaptor molecules, such as MyD88, TLRs lead to the activation of NFκB [12]. By contrast, self-reactive antibodies against self-reactive or cross-reactive DNA co-engage antigen receptors and TLRs, leading to a continuous activation of these auto-reactive B cells and the development of autoimmune disease [12].

Nonetheless, environmental factors are still central to autoimmunity [13]. Indeed, it is possible that the discrepancies in the immune system may lead to infections (which are indeed more frequent in elderly people) thus triggering ADs [14]. Thus, on the one hand, a huge number of microorganisms have been identified as associated with the onset of an overt immune-mediated disease, and on the other hand, patients with ADs are at a major risk for infections [15].

Ramagopalan *et al*. [16] analyzed hospital admissions and death certificates across England, and found that the risk of tuberculosis (TB) is significantly increased in patients with immune-mediated diseases, highlighting the need for TB screening control and treatment policies in these patients. It would be interesting to confirm whether TB is a consequence of the immune system imbalance of the disease *per se*, or a result of the background therapy, or if it represents another causative factor of ADs [17].

It has been shown that a link exists between the mycobacterium, ADs, and, surprisingly, another component of the puzzle, namely, vitamin D [18]. Vitamin D plays a key role as an immunomodulator in autoimmunity [19,20]. This role of vitamin D seems to be so important that ADs show seasonality (due to different levels of UV exposure), and even the month of birth may influence the risk of onset of an AD, as shown by Disanto *et al*. [21]. Moreover, Tincani *et al*. [22], in their review, emphasized that patients affected with Sjögren's syndrome (SS) who have low levels of vitamin D are at a higher risk of developing severe complications such as lymphoma and peripheral neuropathy [23,24]. The need to provide vitamin D supplementation for patients with autoimmune diseases, including SS, is clear. Moreover, the cost–benefit ratio of providing vitamin D as a preventative medicine to the general population should be evaluated, together with the routine monitoring of gestational vitamin D levels [20]. Finally, because (no-longer-just-a-)vitamin D has several physiological properties involved with the innate immune defense against TB, the role of this vitamin in the treatment of TB should be evaluated, especially when associated with autoimmunity [18].

Thus, it is evident that every day, people are exposed to several triggering factors for ADs. People can be exposed to such elements through diet (for example, vitamin D), and other sources. For instance, another defendant, aluminum (alum), is used as an adjuvant in vaccines, and its association with autoimmunity has recently been highlighted [25]. Alum can be viewed as a nanomaterial, that is, a nanocrystalline compound spontaneously forming micron/submicron-sized agglomerates.

Alum has a number of mechanisms by which it works as an adjuvant (Table 1). Moreover, it can be detected within monocyte-lineage cells long after immunization in presumably susceptible individuals who develop systemic/neurologic symptoms. Khan *et al*. [26] showed that in mice, intramuscular injection of alum-containing vaccines was associated with appearance of aluminum deposits in distant organs such as the spleen and brain, where they were still detected 1 year after injection. The chemokine CCL-2 seems to be implicated in systemic diffusion of aluminum particles captured by monocyte-lineage cells, and in the subsequent neurodelivery of these particles.

**Table 1:** **Specific mechanisms relating to the adjuvant properties of aluminum compounds**[28]

**Mechanism**	**Characteristics**
Depot effect	Alum-induced consolidation of the desired antigen at the injection site results in slow release of the antigen to antigen-presenting cells for an extended period of time
Activation of DCs by lipid sorting	Aluminum salts induce activation of DCs and complement components, and increase the level of chemokine secretion in the injection site. Aluminum-based salts firmly bind to and alter the structure of lipids in the plasma membrane of DCs
NLRP3 inflammasome-independent DC activation	Membrane proximal events unrelated to the NLRP3 inflammasome are sufficient to mediate DC activation in response to alum
Augmentation of adjuvant properties of alum by uric acid	Increase in uric acid mediates upregulation of MHC class II in monocytes via an IL-4 dependent mechanism
Host cell DNA	The host cell DNA can enhance the adjuvant properties of alum
Alum-dependent increase in IgE levels	Promotes allergy
Amyloid plaque formation	Alum promotes the formation of amyloid plaques by catalyzingpolymerization of β-amyloid
Increased Tau protein	Alum may increase the levels of Tau protein

Abbreviations: DC, dendritic cell; IL, interleukin; MHC, major histocompatibility complex; NLRP3, nucleotide-binding oligomerization domain-like receptor family, pyrin domain-containing 3.

These various lines of evidence reinforce the idea that alum is neurotoxic, and that continuously escalating doses of this poorly biodegradable adjuvant in the population may become insidiously unsafe, especially in cases of over-immunization or immature/altered blood–brain barrier or high constitutive CCL-2 production.

It is possible that this is part of the explanation for the real new wave in autoimmunity, that of autoimmune/inflammatory syndrome induced by adjuvants (ASIA) [27]. This syndrome comprises four conditions, namely siliconosis, Gulf War syndrome, macrophagic myofasciitis syndrome, and post-vaccination phenomena, which have in common a history of previous exposure to an adjuvant and a group of similar clinical features. Consequently, the importance of discovering a pathogenic role for alum must be emphasized. This compound has been considered a safe adjuvant for 90 years. However, it is now known to be an inducer of dendritic cells and complement activation, which is capable of increasing the levels of chemokine secretion at the injection site and of enhancing the secretion of key T-helper (Th)1 and Th17-cell polarizing cytokines such as interleukin-12 [28]. All these effects lead to development of an autoimmune response. Because many of the symptoms of ASIA could be neurologically dependent, the possibility that alum might traffic to the brain raises questions about its relevance to autoimmune disease. Indeed, the central nervous system (CNS) is still attracting most of the interest of autoimmune scientists, especially with regard to SLE, the disease considered the forerunner of ADs [29].

Neuropsychiatric (NP)SLE is triggered by multiple mechanisms, including several autoantibodies. One of the leading pathogenic autoantibodies in NPSLE is anti-ribosomal P protein. Carmona-Fernandes *et al*. [30] found that the specificity, sensitivity, positive predictive value, and negative predictive value of anti-Rib-P for SLE diagnosis were 99.4%, 14.2%, 90%, and 76.4%, respectively. Although, anti-Rib-P was not clearly associated with any clinical condition, including NPSLE, in that study, nonetheless, the effects of these autoantibodies on the CNS have been addressed in several other studies. One of the most interesting investigations showed that intracerebroventricular injection of an anti-DNA antibody carrying the 16/6 idiotype, which induces the production of anti-Rib-P antibodies, provoked deficiencies in olfactory capabilities and depression in mice [31,32]. Likewise, the 16/16 antibody bound to similar areas in the olfactory machinery as those to which anti-P ribosomal antibodies bind. Kivity *et al*. [33] identified another weapon at the armory of the 16/6 idiotype-expressing antibodies, showing that these antibodies can induce brain inflammation and cognitive impairment in mice. These reports on the effect of this idiotype of the human anti-DNA antibody shed further light on the diverse mosaic pathophysiology of neuropsychiatric lupus, and indicate that there is a possibility of molecularly targeting the disease [34]. This potential for treatment of SLE was addressed by Gono and coworkers [35], who highlighted the possibility that the differences in the cross-reactivity of each autoantibody with the nervous system might explain the diverse clinical features in NPSLE, and that the identification of autoantibody targets could lead to the development of novel therapies; for instance, by protecting specific neuronal cells.

One of the autoimmune conditions more clearly associated with CNS involvement is anti-phospholipid syndrome (APS). Katzav *et al*. [36] have shown that this AD, besides being characterized by the presence of autoantibodies, can be triggered experimentally by coagulopathy. Indeed, this group found that both heterozygous and homozygous Factor V Leiden (FVL) transgenic mice that were immunized with β2-glycoprotein I had significantly higher and longer-lasting immune responses. Furthermore, because these responses were dependent on the FVL mutation allele load, the research suggests that genetically mediated coagulopathies increase the risk of developing coagulation-targeted autoimmune responses. Moreover, raised levels of circulating anti-phospholipid antibodies seem to lead inevitably to neurodegeneration.

The thrombophilic network is also involved in another autoimmune disorder, celiac disease. Lerner *et al*. [37] showed that in addition to the well-known thrombogenic factors, the presence of anti-phospholipid antibodies, namely anti-phosphatidylserine/prothrombin and anti-prothrombin, might play a pathogenic role by increasing the risk of intestinal injury, endothelial dysfunction, platelet abnormality, and enhanced apoptosis. Studies are urgently awaited to address the potential of treating patients with ADs by targeting these antibodies.

A further component in the mosaic of autoimmunity is the question of what stays in the renal pyramids. Renal involvement represents a major issue in autoimmune connective tissue diseases (CTDs). The main question is whether renal damage is caused by the underlying disease or by interventions, particularly by drug reactions as described by Kronbichler and Mayer [38]. Consequently, physicians should always take kidney function into account when treating CTDs [39]. Nonetheless, recent evidence suggests that the real hub for presenting T cells is in the lung [40]. Indeed, T-cell blasts do not enter the CNS efficiently, and they gain this capacity only after residing transiently within the lung tissues. In these tissues, the T cells are stimulated, after which they proliferate strongly, acquire migratory properties by rearranging specific genes and producing targeting chemokines, and can then enter the circulation and induce CNS disease. There are several implications for such phenomena. For instance, the role of smoke as a risk factor in ADs becomes even more important when we consider the exposure of disease-inducing immune cells to tobacco smoke drawn into the lungs. Second, the development of drugs based on the concept of arresting T cells at a hub (that is, in the lung), is a potential therapy and should be encouraged [41].

Phosphodiesterase-targeted therapies 4 are also a promising tool for treating patients with a variety of autoimmune diseases. Intriguingly, these inhibitors have been used to treat both diseases of the CNS (such as Parkinson’s disease) and of the lung (such as chronic obstructive pulmonary disease), suggesting a common soil between the lung and the CNS.

Finally, the revolutionary roads traced by biologic therapies continue to provide benefits for patients with ADs. Rosman *et al*. [42], in their review of the advantages and drawbacks of biologic therapies for autoimmune disease, focused on the importance of having a range of drugs available to treat patients with severe or resistant disease. The important issue here is having a range of different targets, allow personalization of therapy not only for the individual, but also on the basis of the pathogenic mechanisms underlying each specific disorder.

## Conclusion

In conclusion, a new wind is blowing within the branches of autoimmunity, from the unexplored continent of ASIA syndrome [27], which is reaching the arduous banks of personalized medicine and bringing new hope to the leaves of autoimmunity.

## Competing interests

The authors declare that they have no competing interests.

## Pre-publication history

The pre-publication history for this paper can be accessed here:

http://www.biomedcentral.com/1741-7015/11/101/prepub
